# Treatment of Severe Thyrotoxicosis With Therapeutic Plasma Exchange in a Patient With a Methimazole Adverse Reaction

**DOI:** 10.7759/cureus.98315

**Published:** 2025-12-02

**Authors:** Yaroslav Buryk, Concepcion Arteaga, Leah E Magdal, Joseph T DiMarco, Olutomi T Akinsete

**Affiliations:** 1 Division of Pulmonary, Critical Care and Sleep Medicine, University of Miami, Miami, USA; 2 Critical Care, Jackson Health System, Miami, USA; 3 Critical Care, American University of the Caribbean School of Medicine, Miami, USA

**Keywords:** acute management of thyrotoxicosis, acute thyrotoxic crisis, drug-induced thyrotoxicosis, management of thyrotoxicosis, medical critical care, methimazole adverse reaction, severe thyrotoxicosis, therapeutic plasma exchange (tpe), therapeutic plasmapheresis, thyroid-storm

## Abstract

Recent research indicates that therapeutic plasma exchange (TPE) has been successfully used in the treatment of thyrotoxicosis in patients where conventional treatment measures failed to effectively control severe thyrotoxicosis, also known as thyroid storm. We present the case of a woman in her late 70s with a history of atrial fibrillation (AFib), hypertension, hyperthyroidism, and anxiety, who presented to the hospital following a hemorrhagic stroke and myocardial infarction that subsequently developed severe thyrotoxicosis following the administration of levothyroxine. During her treatment course, the patient experienced an adverse reaction to methimazole, complicating management by precluding the use of conventional treatment protocols for severe thyrotoxicosis. TPE was successfully used to stabilize the patient over the course of two plasma exchanges without the need for thyroidectomy or radio-iodine ablation of the thyroid post stabilization, and was able to be safely discharged home.

## Introduction

Thyrotoxicosis is a syndrome caused by an excessive circulation of thyroid hormones, primarily free triiodothyronine (FT3) and/or free thyroxine (FT4). Patients may also show a decreased serum value of thyroid-stimulating hormone (TSH) due to negative feedback by the elevated T3 and T4 hormones [1,2]. To understand these disturbances in thyroid hormones in relation to thyrotoxicosis, it is important to first consider the causes of thyrotoxicosis. This can include disorders that result in hyperactivity of the thyroid gland such as Graves' disease or toxic adenomas. Additionally, external sources such as exogenous thyroid hormone administration can lead to an excess of circulating thyroid hormone leading to a thyrotoxic state [1].

Thyrotoxicosis typically manifests with symptoms including palpitations, heat intolerance, weight loss, anxiety, and proximal muscle weakness [1]. A patient may present with tachycardia, lid lag, tremor, and warm moist skin on physical exam [2]. The symptoms of thyrotoxicosis result from a hypermetabolic state induced by elevated thyroid hormones. Thyrotoxicosis should be managed promptly, or it can potentially lead to the patient developing an emergent condition known as severe thyrotoxicosis or thyroid storm [1, 3, 4]. Thyroid storm is a severe form of thyrotoxicosis distinguished by the rapid exacerbation of symptoms and rapid deterioration within days or hours of onset and is associated with a mortality of 10-30%, even with the use of aggressive medical treatment [5, 6].

First-line treatment involves antithyroid drugs or thioamides, most commonly Propylthiouracil (PTU) or Methimazole (MMI). Both drugs block the enzyme thyroid peroxidase (TPO), preventing thyroid hormone synthesis. PTU can also inhibit thyroid synthesis by preventing the peripheral conversion of T4 to T3 [1,7]. The choice between the two is ultimately dependent on the patient’s clinical state and tolerability [7-11].

However, not all patients respond adequately to thioamides, even when used in combination with corticosteroids, cholestyramine, and beta blockers. In cases of severe refractory thyrotoxicosis as well as thyroid storm, therapeutic plasma exchange (TPE) is a safe and effective second-line alternative to rapidly reduce circulating thyroid hormones [12, 13]. It has even shown strong efficacy in thyrotoxic patients with rapid clinical deterioration or multiorgan decompensation [8, 14-16]. For thyroid storm, the American Society of Apheresis approves of therapeutic plasma exchange being used daily to every three days based on the individual’s symptoms [13]. It is important to note that the use of TPE for thyroid storm is generally a category II therapy. Meaning that it should only be utilized as a second line or adjunctive treatment [13]. The effectiveness of plasmapheresis is determined by the volume of blood being processed, the volume of the plasma exchanged in each process, the frequency of exchange, and other technical features [11, 14, 15].

Since TPE only transiently reduces thyroid hormones, it is generally only used as a bridging therapy prior to thyroidectomy or radio-iodine ablation of the thyroid [12,13]. This bridging therapy is done because the effects of thyroid storm can be so severe that it would prevent the patient from safely undergoing surgery. Consequently, this would eliminate the patient’s endogenous thyroid hormone production and would require the patient to take exogenous thyroid hormone for the remainder of their life.

## Case presentation

**Figure 1:**
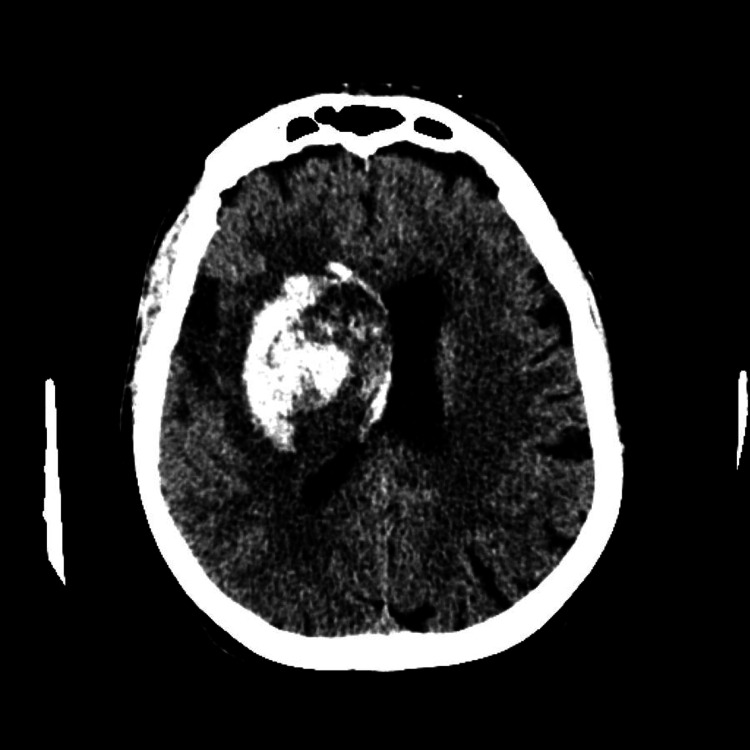
Head CT of right basal ganglia and temporal lobe intraparenchymal hemorrhage with intraventricular hemorrhage and midline shift CT: Computed Tomography

A woman in her late 70s with a past medical history of atrial fibrillation with rapid ventricular rate (RVR), hypertension, hyperthyroidism, and anxiety presented to the hospital with a fixed right-sided gaze, left-sided weakness, and slurred speech. An initial electrocardiogram (ECG) showed normal sinus rhythm with premature atrial contractions, but no acute ischemic changes. Elevated troponin levels were noted. A head computed tomography (CT) scan revealed a right basal ganglia and temporal lobe intraparenchymal hemorrhage with intraventricular hemorrhage and midline shift (Figure 1). Heart failure with reduced ejection fraction (30-35%) was also identified on echocardiography. Her bleed risk was determined to be too elevated to perform either percutaneous intervention or the use of thrombolytics; therefore, she was managed with supportive care. During admission, the patient’s home medications were provided by a family member. Her medications included levothyroxine, clonazepam, clonidine, dicyclomine, fluticasone, montelukast, omeprazole, propranolol, and apixaban. All the medications were continued, except the apixaban due to her elevated bleed risk.

**Figure 2:**
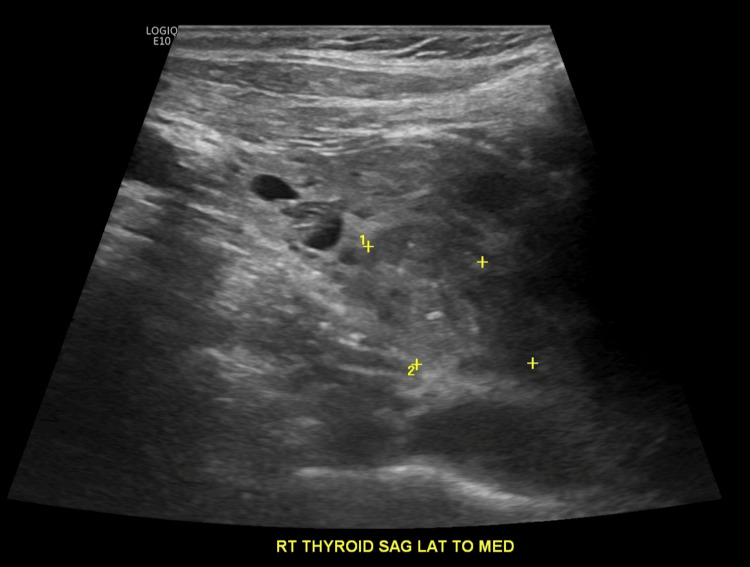
Right thyroid lobe with 2.4cm nodule (labeled 1)

**Figure 3:**
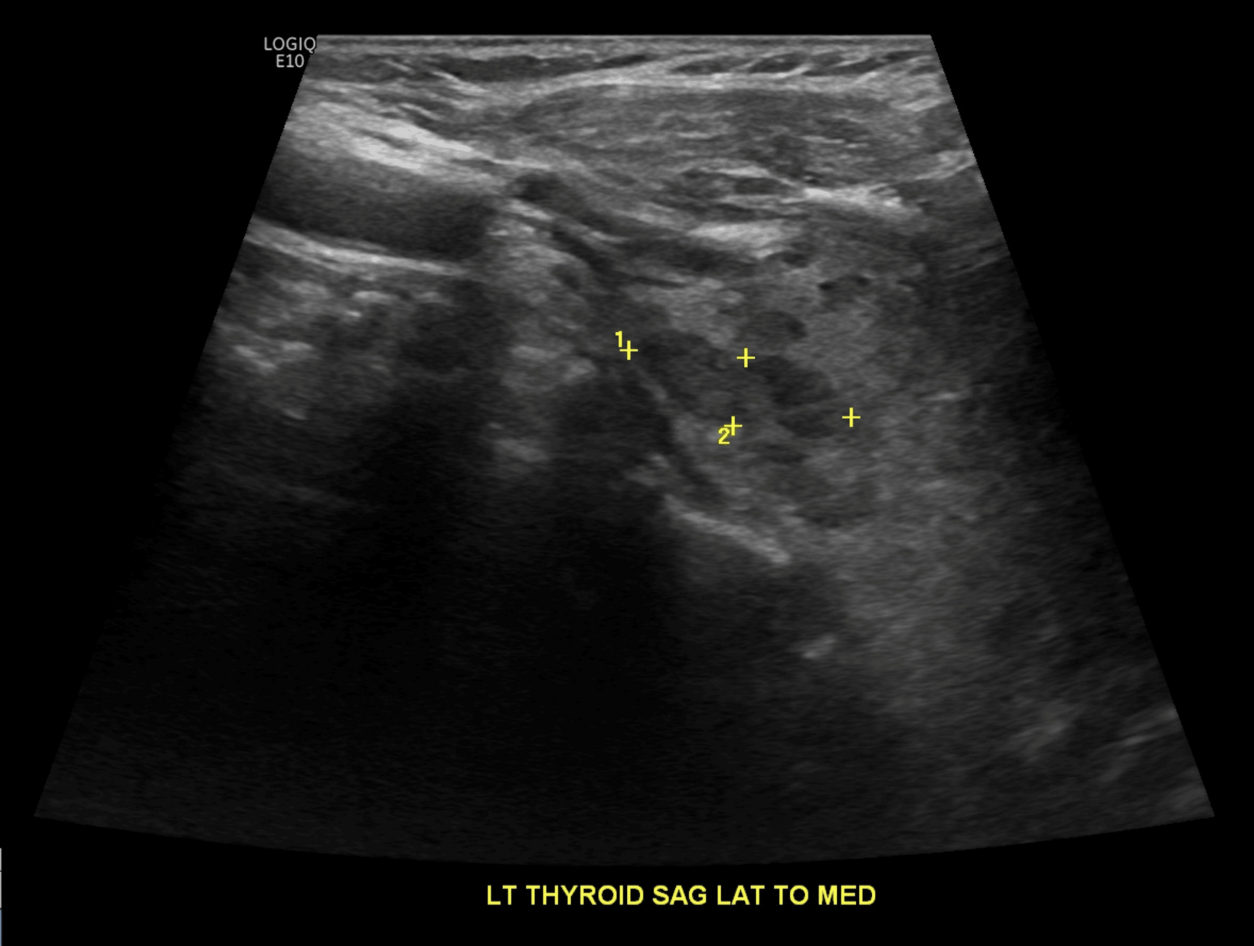
Left thyroid lobe with 1.7cm nodule (labeled 1)

On day 4 of admission, her mental status had improved, and it was discovered that the prescription for levothyroxine had not been current and that she had been noncompliant with her home medications. By this time, the patient had already received three doses of levothyroxine 75mcg, on admission days 2 through 4, before being discontinued. This information, accompanied by the patient's decreased levels of TSH 0.092mU/L, indicated hyperthyroidism. Treatment with methimazole was then started the day after levothyroxine was stopped. Serial thyroid function tests and liver function tests (LFTs) were also ordered (Table 1). Additionally, a thyroid ultrasound was performed and indicated two suspicious nodules (Figures 2, 3) that would require biopsy.

**Table 1:** FT4, AST, ALT, ALP and levels with average heart rate throughout admission. FT4: Free Thyroxine: HR: Heart Rate; AST: Aspartate transferase; ALT: Alanine transaminase; ALP: Alkaline phosphatase

Admission Day	FT4 (0.9-1.7ng/dL)	HR (60-100bpm)	AST (8-49U/L)	ALT (4-36U/L)	ALP (44-147U/L)
1	1.7	86	59	48	97
2	-	93	-	-	-
3	-	85	76	29	87
4	-	78	54	26	80
5	1.99	86	41	27	77
6	-	82	56	41	77
7	1.46	86	-	-	-
8	1.71	89	81	96	112
9	-	90	80	118	106
10	-	97	89	150	105
11	-	94	-	-	-
12	-	97	85	141	110
13	1.85	94	77	132	103
14	-	84	-	-	-
15	2.42	89	58	94	99
16	-	94	-	-	-
17	-	102	40	55	98
18	-	109	35	41	85
19	3.28	104	-	-	-
20	3.76	117	65	72	97
21	3.93	101	52	78	117
22	-	153	-	-	-
23	4.93	137	52	79	142
24	-	140	52	75	110
25	4.51	114	73	115	123
26	4.02	124	61	129	128
27	5.61	120	61	131	154
28	2.13	106	47	54	60
29	2.18	113	44	54	89
30	1.86	125	35	35	54
31	1.95	116	44	47	58
32	2.16	97	68	100	98
33	2.24	101	51	89	85
34	-	112	41	80	92
35	-	90	50	99	132
36	-	89	39	79	99
37	-	70	-	-	-
38	-	85	46	78	-
39	2.45	85	44	67	-
40	-	88	-	-	-
41	-	85	-	-	-

**Figure 4:**
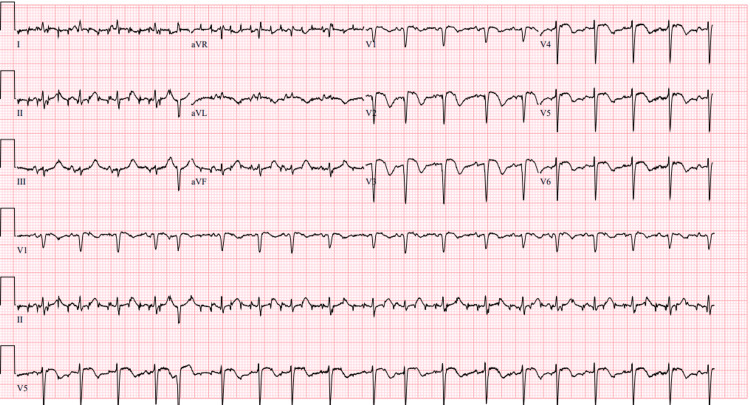
Atrial fibrillation with rapid ventricular rate

After four days of treatment with methimazole, her LFTs became elevated to an AST of 89U/L and ALT of 150U/L. Methimazole was halted due to the concern of hepatotoxicity. An adverse reaction to methimazole was suspected, and thyrotropin receptor antibody (TRAb) and thyroid-stimulating immunoglobulin (TSI) tests were ordered and were found to be negative. Five days after stopping methimazole, TSH levels decreased to below 0.0150mU/L and FT4 levels were slightly elevated to 2.42ng/dL. Due to the patient’s elevated FT4 level and medical history, treatment was initiated with prednisone, cholestyramine, and metoprolol. These medications were started three days after discontinuing methimazole. Even with these medication adjustments, the patient's mental status declined. A head CT scan revealed the original bleed had worsened and was managed supportively. Four days after starting the prednisone, cholestyramine, and metoprolol, the patient’s FT4 levels had further increased to 3.28ng/dL. She had also met the diagnostic criteria for thyrotoxicosis according to the Burch-Wartofsky Point Scale (BWPS), with her score being over 45. Methimazole was reattempted a total of eight days after originally stopping the methimazole, and at that time her LFTs had returned to within normal range, but was unfortunately halted after a single dose due to another surge in her LFTs to an AST of 65U/L and ALT of 72U/L. PTU 25mg twice per day was then initiated five days after stopping methimazole, but her symptoms continued to worsen, with the most serious concern being the onset of a new episode of atrial fibrillation with rapid ventricular rate (AFib with RVR) (Figure 4). The day after her AFib with RVR started, PTU was changed to once a day, and Propranolol was initiated. The patient was moved to a critical care unit for treatment of thyroid storm.

**Figure 5:**
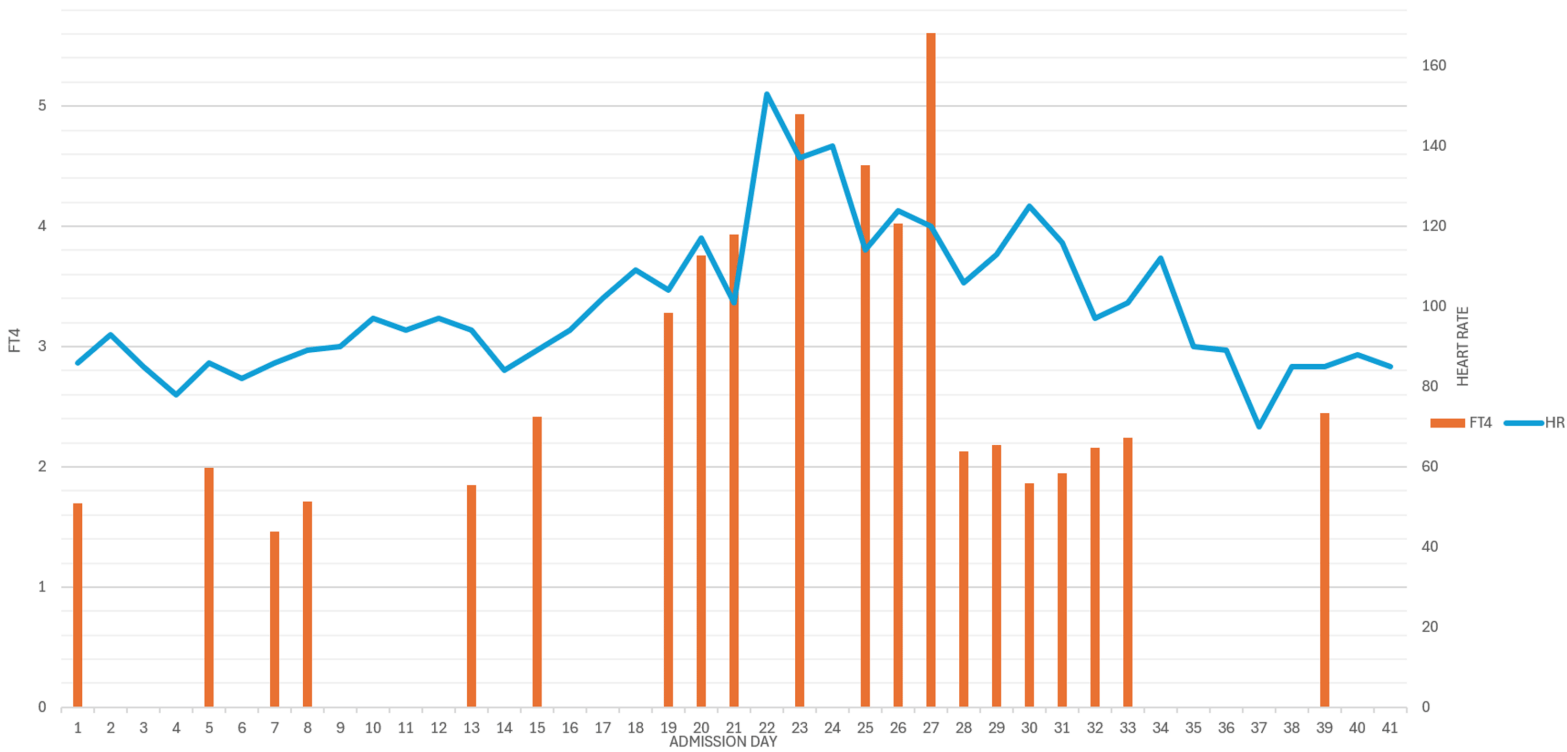
Average heart rate compared to free thyroxine levels throughout admission FT4: Free Thyroxine (ng/dL) HR: Heart Rate (beats/minute)

After four days of medical therapy with PTU, propranolol, and prednisone, the AFib with RVR remained uncontrolled, and her mental status deteriorated. The first session of TPE was initiated via a femoral central line. The initial session decreased her FT4 level from 5.61ng/dL to 2.13ng/dL. The second TPE session was done two days later and further reduced her FT4 from 2.18ng/dL to 1.86ng/dL (Figure 5). These sessions were performed on days 27 and 29 (Table 1). Four days after the second session of TPE, her heart rate was able to be controlled to normal sinus rhythm, and the central line was removed the following day. She was then downgraded from the critical care unit and kept for observation for another eight days. She had no return of symptoms and only a mildly elevated increase in her FT4 of 2.45ng/dL. An outpatient appointment was made with her endocrinologist to monitor thyroid hormone levels, schedule a biopsy of the thyroid nodules, and determine an optimal plan of care. After remaining asymptomatic for eight days post-stabilization, she was discharged home with prescriptions for PTU, propranolol, and prednisone and instructed to follow up with an endocrinologist within one week of discharge.

## Discussion

Thyrotoxicosis can develop into a life-threatening condition known as thyroid storm if not appropriately managed. Thyroid storm requires immediate intervention and critical care, with a reported mortality rate of 10-30% even with timely treatment [8, 9, 15-18]. Key indicators of thyroid storm include elevated thyroid hormone levels, tachycardia, hyperthermia, and neurologic dysfunction. The Burch-Wartofsky Point Scale (BWPS) remains a useful diagnostic tool in these cases [9]. Severe thyrotoxicosis is typically managed with a combination of therapies, including propranolol, thioamides (methimazole or propylthiouracil), iodine, cholestyramine, and glucocorticoids. Definitive treatment, such as thyroidectomy or radio-iodine ablation, is generally reserved for refractory or recurrent cases [7]. TPE has previously been described to be used for thyroid storm as a bridging therapy in the case of patients who were either unable to tolerate thioamides or done prior to emergent thyroidectomy or radio-ablation of the thyroid [19-20].

This case describes a patient with medication-induced thyrotoxicosis complicated by the presence of hemorrhagic stroke, myocardial infarction, and an adverse reaction to methimazole. This patient's cause of thyrotoxicosis was due to inappropriate use of levothyroxine. In this case, the patient's AST levels increased from 56 U/L to 81 U/L and ALT levels increased from 41 U/L to 96 U/L within 48 hours of methimazole treatment on days six through eight of admission and trended upward until therapy was halted, indicating a potential for hepatotoxicity (Table 1). Although it has been documented that myocardial infarctions have been shown to transiently increase AST and ALT levels, this patient received a single-dose trial with methimazole on day 18 of admission after the previous AST and ALT had returned to normal levels following the cessation of methimazole for eight days. Following the administration of methimazole, there was a subsequent increase in AST levels from 35 U/L to 65 U/L and ALT levels from 41 U/L to 72 U/L within 48 hours. This rapid increase indicated a true adverse reaction and limited the possibility of aggressive use of thioamides.

The elevation of liver enzymes is a rare but known potential side effect for either methimazole or PTU and was the only adverse reaction observed. This led to the subsequent reduced dose of PTU, 25mg given daily, which is below what current guidelines indicate for thyroid storm treatment. This conservative approach was done to prevent and limit any potential adverse effects that could be caused by PTU, as it has been shown that patients who have had an adverse reaction to methimazole are at an increased risk for an adverse reaction from PTU [19].

This led to the use of TPE to stabilize the patient's hyperthyroid state. TPE works by filtering out and replacing the patient's plasma, which will remove T3 and T4 that are both free and bound to albumin and thyroid-binding globulin, as well as catecholamines and cytokines present in circulation. This allows new unbound albumin for the free thyroid hormones to bind, which will further reduce circulating levels of T3 and T4 [18]. This treatment typically takes three to six sessions to decrease thyroid hormone levels enough to achieve clinical stabilization and normalization of thyroid hormone levels. This patient was able to achieve full resolution of hyperthyroid-associated symptoms four days after completing TPE.

The conventional approach for the use of TPE to treat thyroid storm is as a bridging therapy to stabilize the patient prior to either thyroidectomy or radio-iodine ablation of the thyroid, as the effects of TPE are transient [4, 13, 18]. This patient was able to be stabilized without return of symptoms and was able to be discharged home without undergoing subsequent removal or ablation of endogenous thyroid function. In this case, a mild elevation of FT4 to 2.45 U/L was observed without the return of symptoms eight days after the final session of TPE, and TSH remained below 0.0150 mU/L, which had been unchanged since day 9 of admission. In this case, thyrotoxicosis was induced by levothyroxine, and at the time of discharge, had been halted for 36 days. It was deemed that the patient could be safely discharged home with family to follow up with Endocrinology within a week of discharge for long-term monitoring and management of thyroid function and biopsy of the thyroid nodules. Additionally, the caretakers were counseled on the signs and symptoms of thyrotoxicosis and instructed to return if symptoms began. Although this case was complicated by the failure of conventional therapy, it serves as an example of TPE being used to successfully treat severe thyrotoxicosis induced by the improper use of levothyroxine.

## Conclusions

Not all patients will respond effectively to the standard medications used to treat elevated thyrotoxicosis. In this case, the patient’s severe thyrotoxicosis was induced by levothyroxine and was complicated by age, comorbid conditions that were both acute and chronic, and an adverse reaction to methimazole that prevented the use of conventional treatment. This case demonstrates how TPE was used to stabilize the patient’s severe thyrotoxicosis without thyroidectomy or radio-iodine ablation of the thyroid following TPE. Clinical stabilization was achieved with two sessions of TPE performed two days apart with full resolution of symptoms occurring four days following the final session of TPE. The patient remained clinically asymptomatic for eight days following the final session of TPE until being discharged. Although mild elevations of FT4 were observed following TPE, the highest recorded level was 2.45 ng/dL which was recorded six days following the final TPE session with the patient remaining asymptomatic. This case highlights a case where TPE was used to safely and effectively treat a patient suffering from severe thyrotoxicosis induced by levothyroxine that was unable to be controlled with standard medical treatment without requiring emergent surgical intervention in an acute setting.
